# Mandragora

**Published:** 1889-09-21

**Authors:** 


### MANDRAGORA.

"Poppy and mandragora" were once classed together among the "drowsy syrups of this world," but while poppy juice has been employed in ever-increasing strength and variety as a narcotic—has even given the generic name of "opiate" to all sleep-producing drugs—mandragora has been allowed to fall into desuetude. Yet it is interesting as having been the first anæsthetic known to mankind. After the thirteenth century it does not seem to have been used. For six centuries, till Simpson brought chloroform into use, mankind endured the ills that flesh is heir to, either from disease, accident, or the surgeon's knife, without an anodyne of any sort. But the old Greek doctors, Dioscorides and the rest, habitually administered it to their patients before subjecting them to any operation, either by the knife or by actual cautery. Dioscorides says: "A wine is prepared from the bark of the root without boiling, and three pounds of it are put into a *cadus* (about eighteen gallons) of sweet wine, and three *cyathi* (a *cyathus* equalled about an ounce and a half, fluid measure) of this are given to those who require to be cut or cauterised; when being thrown into a deep sleep, they do not feel any pain."

Pliny also speaks of it, not merely as an anæsthetic, but as a drug which had the power to produce the semblance of death while life still existed. As the action of mandragora is to deaden sensation in the nervous centres, and as it also interferes with respiration a considerable time before the action of the heart ceases, even when given in doses likely to be fatal, this appearance of death might easily deceive an ordinary observer. It would be, indeed, difficult to believe that life still existed in the cold, pulseless body. It is easy to find an all but strictly accurate account of the action of mandragora:

> "Take thou this phial, being then in bed,
> And this distilled liquor drink thou off;
> When, presently, through all thy veins shall run
> A cold and drowsy humour; for no pulse
> Shall keep his native progress, but surcease,
> No warmth, no health, shall testify thou liv'st;
> The roses in thy lips and cheeks shall fade
> To paly ashes; thy eye's windows fall
> Like death when he shuts up the day of life;
> Each part, deprived of supple government,
> Shall, stiff, and stark, and cold, appear like death;
> And in this borrowed likeness of shrunk death,
> Thou shalt continue two and forty hours,
> And then awake as from a pleasant sleep."

This is Friar Lawrence's address to Juliet when he gives her the medicine which is, by apparently killing her, to save her from being married to Paris. The last line alone is inaccurate. From the anæsthesia produced by mandragora, as from that produced by most other drugs, one does not awake "as from a pleasant sleep," but in a state of increasing terror akin to delirium. The visions that assail Juliet before she drinks the potion are much more akin to the real after-effects of mandragora, but it is natural enough that the friar would not frighten Juliet by telling her the real mood in which she would wake. The madness that resulted from the use of mandragora was so strange and characteristic that those who suffered from it were called mandrakes or mandragorites. As the drug went out of use, the tradition of this madness became applied to the plant itself :

> " And shrieks like mandrakes torn out of the earth,
> That living mortals, hearing them, run mad."

Another use to which mandragora was put was akin to its employment in medicine. A preparation of it called "morion," or death wine, was administered to criminals who were condemned to torture, or to a lingering and painful death. It is a curious instance of the reaction of human instinct from the laws approved by the ideas of justice existing in ancient times, that judges would first pronounce a cruel sentence, and then permit this amelioration of its pain. It was, in all probability, this "morion" which was offered to Christ on the cross. Indeed, it was customary, when the Romans had introduced death by crucifixion, for Jewish women to administer this morion to the sufferers. This not only deadened suffering, but caused the sleep that simulated death. It became known to the authorities that in many cases a criminal, apparently dead, recovered after being taken down from the cross, and they then gave orders that the bodies of the crucified should be mutilated before the friends were allowed to take them away for burial. In this custom we have the explanation of the spear thrust into Jesus' side, and the breaking of the thieves' legs.

It is doubtful if mandragora will ever take its old place in the pharmacopoeia. We have many narcotics now—perhaps too many. But besides its general narcotic quality, it has a powerful local anæsthetic effect, which might make it valuable in minor surgery.

